# Transcriptome and metabolome profiling of the medicinal plant *Veratrum mengtzeanum* reveal key components of the alkaloid biosynthesis

**DOI:** 10.3389/fgene.2023.1023433

**Published:** 2023-01-20

**Authors:** Jiajia Liu, Lijun Han, Guodong Li, Aili Zhang, Xiaoli Liu, Mingzhi Zhao

**Affiliations:** ^1^ College of Chinese Materia Medica, Yunnan University of Chinese Medicine, Kunming, China; ^2^ Yunnan Key Laboratory for Dai and Yi Medicines, University of Chinese Medicine Kunming, Kunming, China; ^3^ Kunming Medical University Haiyuan College, Kunming, China

**Keywords:** Veratrum spp., alkaloid biosynthesis, medicinal plants, liliaceae, gene expression, alkaloid profiling

## Abstract

*Veratrum mengtzeanum* is the main ingredient for Chinese folk medicine known as “Pimacao” due to its unique alkaloids. A diverse class of plant-specific metabolites having key pharmacological activities. There are limited studies on alkaloid synthesis and its metabolic pathways in plants. To elucidate the alkaloid pathway and identify novel biosynthetic enzymes and compounds in *V. mengtzeanum*, transcriptome and metabolome profiling has been conducted in leaves and roots. The transcriptome of *V. mengtzeanum* leaves and roots yielded 190,161 unigenes, of which 33,942 genes expressed differentially (DEGs) in both tissues. Three enriched regulatory pathways (isoquinoline alkaloid biosynthesis, indole alkaloid biosynthesis and tropane, piperidine and pyridine alkaloid biosynthesis) and a considerable number of genes such as *AED3-like*, *A4U43*, *21 kDa protein-like*, *3-O-glycotransferase 2-like*, *AtDIR19*, *MST4*, *CASP-like protein 1D1* were discovered in association with the biosynthesis of alkaloids in leaves and roots. Some transcription factor families, i.e., *AP2/ERF*, *GRAS*, *NAC*, *bHLH*, *MYB-related*, *C3H*, *FARI*, *WRKY*, *HB-HD-ZIP*, *C2H2*, and *bZIP* were also found to have a prominent role in regulating the synthesis of alkaloids and steroidal alkaloids in the leaves and roots of *V. mengtzeanum*. The metabolome analysis revealed 74 significantly accumulated metabolites, with 55 differentially accumulated in leaves compared to root tissues. Out of 74 metabolites, 18 alkaloids were highly accumulated in the roots. A novel alkaloid compound viz; 3-Vanilloylygadenine was discovered in root samples. Conjoint analysis of transcriptome and metabolome studies has also highlighted potential genes involved in regulation and transport of alkaloid compounds. Here, we have presented a comprehensive metabolic and transcriptome profiling of *V. mengtzeanum* tissues. In earlier reports, only the roots were reported as a rich source of alkaloid biosynthesis, but the current findings revealed both leaves and roots as significant manufacturing factories for alkaloid biosynthesis.

## 1 Introduction

Plants usually produce numerous specialized metabolites involved in growth, development, and defense *via* specialized and primary metabolic pathways (Mithöfer and Boland, 2012; Mizutani, 2012). These natural products or phytochemicals play prominent roles in protection, survival, and defense against different stresses (Zaynab et al., 2018; Khare et al., 2020; Jan et al., 2021). According to the world health organization (WHO), approximately 65%–80% of the global population living across developing countries solely rely on plant sources as primary healthcare (Calixto, 2005; Tomlinson and Akerele, 2015). In China, the tradition of using herbs for treating various diseases has been retained since ancient times and has been an effective practice for treating diseases before widely accepted modern medicine. For the people living in remote areas, it has been the primary way of treating various diseases and still holds a prominent position in their daily healthcare and living and its value in quantitative terms remains explorable. However, the fast expansion of China’s social economy threatens the long-term use of traditional medicinal herbs. However, some regions have recorded and maintained the basic knowledge and information source about traditional medicinal plants. It is still limited and needs more attention to document knowledge about the traditional medicinal plant, its usage, urgent conservation efforts and specifically on different biochemical and molecular mechanisms which are still hidden from the world (Au et al., 2008; Li and Xing, 2016).

While considering natural resources as the source of medicines, plant-synthesizing alkaloids that are nitrogen-containing and have been proven as the prominent source through the pharmaceutical industry, especially steroidal alkaloids, are the most vital. So far, the members of the Solanaceae Apocynaceae and Buxaceae families are the most essential and significant sources of these alkaloids, which may be due to their structural similarities with mammalian steroid hormones having therapeutic effects (Cordell, 1998; Jiang et al., 2005). The genus *Veratrum* is one of the most important members of the Liliaceae (Melanthiaceae) family and almost 17 to 47 perennial herbaceous plant species are enlisted in it (Zomlefer et al., 2003; Zomlefer et al., 2018). It is reported that members of this family have been extensively found in the northern hemisphere in temperate to arctic zones and most of them are said to have originated in East Asia (Fang et al., 2005; Zomlefer et al., 2018). In China, almost 13 specie and one variety have been reported and used in medicines for 1700 years and the most prominent among them are *V. maackii, V. schindleri, V. nigrum*, and so many others (Chinese Herbal Dictionary, 2006). Primarily, *V. mengtzeanum* has been used as the primary source of the most important Chinese folk medicine known as “Pimacao,” for treating rheumatic pain, wound hemostasis and bruises (Chinese Herbal Dictionary, 2006; Yin et al., 2014). Besides this, it has a superior economic value and is considered the principal constituent drug of the Yilizhitong pill and Yunnan Baiyao (Yin et al., 2014).

The particular interest of species in the *Veratrum* genus lies in the therapeutic and medicinal properties of steroidal alkaloids found in it. Almost 100 alkaloids were reported from this genus, most of which were extracted from roots and rhizomes. Many alkaloids have been demonstrated to possess therapeutic effects in the induction of bradycardia, analgesia, cancer suppression, and several other effects (Chandler and McDougal, 2014b). The major steroidal alkaloids isolated from the *Veratrum* plant have pharmacological properties, including controlling blood pressure, anti-thrombosis, anti-inflammatory, antiplatelet aggregation, analgesic, and antitumor activities (Zomlefer et al., 2003). The plant-based steroid alkaloids also participate in anti-diabetic activities of human body *via* control of complications related to diabetes without any side effects. These alkaloids from several species of *Veratrum* genus have been utilized as insecticides besides their use as medicine for long. The roots of these plants were previously used to add toxicity to the sneezing powder. This is due to the reason that the flowers and bulbs of *Veratrum* contain toxic alkaloids, but they rarely cause toxicity to human body (Matsuura and Fett-Neto, 2015; Dirks et al., 2021).

Previous research employing radiolabeled precursors has resulted in biochemical elucidation in the initial steps during alkaloid biosynthesis (Pelletier, 1983). Despite their diverse structures, all steroidal alkaloids are produced from the same core intermediate, norbelladine, through condensing tyramine with 3,4-DHBA. There are many similarities among several metabolic pathways specified in plants. The initial step in alkaloid biosynthesis is achieved by coupling two different available precursors that define the entrance point for the primary metabolites towards the specific pathways involved in alkaloid biosynthesis—combining tyramine and 3,4-DHBA results in developing a Schiff base intermediate that goes further through reduction and formulates norbelladine (Pelletier, 1983; Brossi, 1985). Furthermore, cyclizing 4′-O-normethylbelladine is carried through three distant procedures, including intramolecular C-C-oxidative-phenol-coupling, which could be termed “para-para,” “para-ortho,” and “ortho-para” resulting in the generation of three main structures. These backbones are the different basis for the formation of alkaloid diversity. It is reported that a complex network exists across enzymatic reactions for producing various compounds across various species, varieties, and cultivars, even across tissues within the same plant. Such biochemical modifications can usually be attained through various enzymes catalyzing different reactions, including demethylations, hydroxylations, C-C O- and N-methylations, formation of C-O bonds and oxidation-reduction reactions (Yamada and Sato, 2013).

There is limited knowledge available for the genes involved in alkaloid synthesis in various species, and there are preliminary reports regarding genes corresponding to alkaloid synthesis. Recently, transcriptome analysis coupled with metabolome is becoming a popular tool for discovering novel genes that encode such types of proteins that actively participate in the biosynthesis of alkaloids (Desgagné-Penix et al., 2012; Hagel et al., 2015). Specialized RNA sequencing technology based on Illumina sequencing is currently extensively used to analyze different medicinal plant transcriptomes. It has helped elucidate numerous specific metabolic pathways (Hao et al., 2011; Gahlan et al., 2012; Hao et al., 2012). For example, several new transcripts showing associations across terpenoid-indole alkaloids in *C. roseus* have been discovered (Rischer et al., 2006). Furthermore, comparing transcriptome profiles with metabolic data enabled researchers to understand better the biosynthesis of various anthraquinones, camptothecin and especially anti-cancerous alkaloids (Yamazaki et al., 2013).

Until now, there is no reported research on the *V*. *mengtzeanum* genome, an annual herb endemic to southwestern China that belongs to the genus *Veratrum* (Liliaceae). Current research was carried out to elucidate the alkaloid pathways and discover novel associated transcripts and metabolites through transcriptome and metabolite profiling across 2 *V*. *mengtzeanum* tissues. Such novel resources may allow insight into the assembly of a biochemical snapshot that represents alkaloid metabolism and guide towards elucidating pathway as well as efforts for searching novel biosynthetic enzymes within such an important medicinal plant.

## 2 Materials and methods

### 2.1 Sample collection

Sample collection for the current study was carried out from Dasecong Village, Secong Village Committee, Datong Sub-district Office, Shizong County, Qujing City, Yunnan Province, 1860 m above sea level (24°45′2.08″N; 103°59′19.06″E) during April 2021. The soil samples were also collected along with plant samples. The soil analysis of the samples revealed that the texture was alluvial with gray color and physio-chemical properties “salt content (14.63 mgg^−1^), electrical conductivity (EC) (4,088 µScm^−1^), Ca^2+^ (1.776 mgg^−1^), K^+^ (0.265 mgg^−1^), Mg^2+^ (0.182 mgg^−1^), Cl^¯^ (0.537 mgg^−1^), Na^+^ (2.473 mgg^−1^), SO_4_^2¯^ (8.49 mgg^−1^) and pH 8.03". Two sample groups containing leaves and roots from three plants (biological replicates) were selected to obtain sample tissues washed with fresh water just after collection and stored at −80°C.

### 2.2 RNA isolation and transcriptome sequencing and analysis

Preparation and sequencing of 6 mRNA libraries on HiSeq 2000 (Illumina) were performed after extracting total RNA from leaves and root tissues of *V. mengtzeanum* Loes using TRIzol reagent. The main steps included purification of RNA samples, double-stranded cDNA synthesis, joint-addition, DNA library amplification cum quality detection, and a few more steps followed by transcriptome sequencing. Then, the quality of libraries was inspected with the help of software named Qubit 2.0 and an Agilent 2,100 bioanalyzer was used to detect specific insert sizes. Following this inspection, different libraries were pooled, and sequencing was done based on Sequencing technology known as Sequencing by Synthesis (SBS) technology using Illumina-HiSeq-high-throughput-sequencing-platform. Furthermore, cleaned reads were calculated for “Q20, Q30 and GC” contents.

The raw reads obtained from sequencing were then filtered for getting clean as well as high-quality reads by eliminating low-quality bases using threshold Q-value ≤20, poly-N > 10% coupled with adaptors, and were performed with the help of software package “fastp” (v 0.18.0) (Chen et al., 2018). Simultaneously, GC contents, Q20 and sequence duplication levels in clean data have been assessed. Total clean reads were assembled into unigenes through Trinity software (v 2.6.6). Moreover, gene expression levels were estimated using the RSEM software package (v1. 2.12) (Li and Dewey, 2011) from RNA-seq data. This software also gave the FPKM (fragment/kb of transcript/million mapped reads) values for each transcription region to estimate their variation and abundance of expression. The threshold value of false discovery rate (FDR) for significance in the observed differential gene/transcript expression has been considered as < 0.05 with absolute values of log_2_FC ≥ 1.

The differential expression of RNAs between two groups of samples was assessed with the help of DESeq2 (Love et al., 2014). To get complete information regarding the annotation of genes’ functions, several different databases were required and, thus, consulted, such as databases related to genome, sequence, gene functions, protein as well as protein interactions. In the current study, the sequences of unigenes, as well as differentially expressed genes (DEGs), have been compared by using Gene Ontology (GO) (Ashburner et al., 2000), Kyoto encyclopedia of genes and genomes (KEGG) (Kanehisa and Goto, 2000), Protein family (Pfam), NCBI non-redundant protein sequences (Nr), and euKaryotic Ortholog Groups (KOG), databases using BLAST software to identify the significant differences among genes based on GO terms and regulatory pathways. The transcription factors (TFs) annotations were predicted by iTAK software by integrating two databases, i.e., PlnTFDB and PlantTFDB. Their working principle was based on the TF family and its identification with the help of means can provide a comparison.

### 2.3 Network analysis

The identified DEGs were further scrutinized while investigating their protein-protein interactions with the help of String v10 (Van der Auwera et al., 2013). It has generated networks of hub-genes harboring nodes and lines for revealing genes and interactions amongst them, respectively. The resultant files comprising these networks were visualized with the help of Cytoscape (v3.7.1) software (Szklarczyk et al., 2015).

### 2.4 Sample extraction and metabolome profiling

The sample preparation to extract and quantify metabolites was performed by MetWare Co., Ltd. (Chen et al., 2013). Approximately 100 mg of freeze-dried vacuum leaves and root tissues of *V. mengtzeanum* were utilized. The fine powder was dissolved in 1.0 mL methanol (70%) by vortex for 30min for 30s each time and kept overnight at 4 °C. Then, after using a centrifuge at 12,000rpm with a time of 10min, extracts went through filtering (0.22 µm pore size) and were analyzed *via* UPLC-MS/MS platform (UPLC, SHIMADZU Nexera X2, www.shimadzu.com.cn/; MS/MS) (4500 QTRAP, http://sciex.com/) (Chen et al., 2009). The qualitative analysis was accomplished according to secondary spectral information based on the self-built database MWDB (metware database) and isotope signal, the repeated signal containing K^+^, NA^+^, NH^4+^, and other large molecular weight substances were removed during the analysis. The process of mass spectrometry yielded data that was analyzed by Analytic 1.6.3. Metabolite quantification was done using triple and quadruple mass spectrometry through multi-reaction monitoring (MRM) analysis. LIT and triple quadrupole (QQQ) scans were developed on a triple quadrupole linear ion trap mass spectrometer (Q TRAP). The metabolite data were analyzed *via* Principal component analysis (PCA), orthogonal partial least squares discrimination analysis (OPLS-DA), cluster analysis and Pearson’s correlation analysis using the R software package *MetaboAnalystR* (Chong and Xia, 2018). The metabolites identified through them were subjected to the OPLS-DA model (Thévenot et al., 2015); then, the metabolites with fold change >2 or <0.5 and variable importance in projection (VIP) values > 1 were taken as differential metabolites for the discrimination of treatments and control groups. Moreover, the KEGG pathway database (http://www.kegg.jp/kegg/pathway.html) (Kanehisa and Goto, 2000) was utilized for the classification and pathways enrichment analyses related to differentially accumulated metabolites (DAMs) to determine their related key pathways.

### 2.5 Candidate genes validation *via* quantitative real-time PCR

For the validation of transcriptome data, the widely accepted technique of qRT-PCR was utilized. For the said purpose, the same RNA samples from leaves and root tissues were operated, which were used for transcriptome analysis (RNA-seq). Some random genes with varying expression patterns in leaves and root tissue samples were selected for the quantitative real-time PCR analysis. The protocol and conditions followed for qRT-PCR analysis, provided in Supplementary files, were done using 2× SYBR Green PCR Master (Roche, Germany) and a Service kit (Beijing, China). The reaction performed was followed by melt curve analysis to ensure the primers’ specificity. For the calculation of expression level related to each sample used for analysis, using the 2^¯ΔΔCT^ method (Livak and Schmittgen, 2001).

### 2.6 Conjoint analysis

The systematic and comprehensive integrated statistical analyses of transcriptome and metabolome data (Bouhaddani et al., 2016) for *V. mengtzeanum* leaves and root biomass were conducted to establish the relationships between genes and metabolites in leaves and roots. It was done *via* a combination of biological functional analyses, correlation analysis, metabolic regulatory pathways and function annotation analyses to screen out key genes or metabolic regulatory pathways involved in alkaloid biosynthesis. Such genes related to alkaloids and steroid alkaloids biosynthesis pathways have been selected for this analysis. Batch data after normalization were used for the analysis *via* R software in the “*cor”* package (González et al., 2008). Pearson’s correlation coefficient *R* ^2^ ≥ 0.8 with *p*-values ≤0.05 was used for the correlation analysis and corrected for the Bonferroni multiple tests.

## 3 Results

### 3.1 Illumina sequencing and assembly

The current study utilized leaves and roots samples of *V. mengtzeanum* with three biological replicates each. Thus, six samples underwent transcriptome sequencing based on the Sequencing by Synthesis (SBS) technology of Illumina HiSeq high-throughput sequencing. It yielded a total of 43.35 Gb clean data, with each sample having clean data of around 6 Gb and a base percentage of 94% as Q30 or more (Supplementary Table S1). A summary of clean reads data used in the subsequent analyses are given in Table 1. Several 190,161 unigenes’ sequences were assembled with N50 of 844bp and an average length of 668bp (Supplementary Table S2).

**TABLE 1:** List of highly accumulated steroidal alkaloids in leaf and root samples of *V*. *mengtzeanum*.

Index	Compounds	Root	Leaf	Type	Found in other *Veratrum* species
Cmlp005477	Veramadine A	4.94 E+07	2.29 E+07	down	Tanaka et al. (2011)
Cmlp005885	3-Vanilloylygadenine	3.90 E+07	1.60 E+07	down	
Cmlp003387	6,7-Epoxyverdine	3.01 E+07	1.15 E+07	down	Chandler and McDougal, (2014b)
Cmlp005383	Germidine	2.31 E+07	4.03 E+06	down	Li et al. (2012)
					Chandler and McDougal, (2014a)
					Senthilkumaran et al. (2015)
Cmlp005589	Veramiline-3-O-β-D-glucopyranoside	2.23 E+07	8.46 E+06	down	Mizuno et al. (1990)
					Zhou et al. (2003)
					Sun et al. (2012)
Cmlp006889	Germanitrine	2.17 E+07	3.14 E+06	down	(Kupchan and Afonso, 1959
					Li et al. (2012)
Cmlp005973	Stenophylline D	2.00 E+07	3.93 E+06	down	Mizuno et al. (1990)
					Zhou et al. (2003)
					Li et al. (2012)
Cmlp006434	Verabenzoamine; 3-veratroyl-15-methylbutyrylgermine	1.72 E+07	7.33 E+06	down	Li et al. (2012); Chandler and McDougal, (2014a); Li et al. (2020)
Cmlp006709	Verazine	1.36 E+07	5.97 E+07	up	Li et al. (2012)
					Augustin et al. (2015)
					Anwar et al. (2018)
					Li et al. (2020)
					Meng-Zhen et al. (2020)
Cmlp004157	Pseudojervine	1.33 E+07	4.63 E+07	up	Krayer (1949)
					Kupchan and Suffness (1968)
					Shakirov and Yunusov, (1971)
Cmlp005636	Veratramine	4.57 E+05	7.46 E+07	up	Krayer (1949)
					Li et al. (2012)
					Anwar et al. (2018)
					Li et al. (2019b)
					Kim et al. (2020)
					Meng-Zhen et al. (2020)
Cmlp006572	Epiverazine	1.31 E+07	6.02 E+07	up	Li et al. (2012)
					Meng-Zhen et al. (2020)
Cmlp005994	Shinonomenine	2.94 E+06	4.85 E+07	up	Kaneko et al. (1978)
Cmlp006270	Hosukinidine	1.31 E+07	4.64 E+07	up	Kaneko et al. (1979)
Cmlp005711	Solanidine	4.49 E+06	3.89 E+07	up	(Pelletier and Jacobs (1953)
					Storey (2007)
					Meng-Zhen et al. (2020)
Cmlp007378	Escholerine	4.25 E+06	2.98 E+07	up	(Morris Kupchan and Ian Ayres (1959)
					Chandler and McDougal, (2014a)
Cmlp005432	Jervine	9.38 E+05	2.90 E+07	up	Krayer (1949)
					Kupchan and Suffness (1968)
					Omnell et al. (1990)
					Suladze et al. (2006)
					Li et al. (2012)
					Dumlu et al. (2019)
					Zheng et al. (2019)
					Meng-Zhen et al. (2020)
					Melnik et al. (2022)
Cmlp002597	Verdine	1.62 E+07	1.58 E+07	up	Li et al. (2012)

### 3.2 Gene annotation and functional classification

Out of 190,161 unigenes, 66,013 were annotated for at least one database. In particular, 49,309 unigenes got annotation in the GO database, 47,207 unigenes were annotated using the KEGG database; 62,090 unigenes got annotation in the Nr database; 40,614 unigenes in the Swissprot database; 61,704 in Tremble, 42,618 unigenes in KOG and 46,329 unigenes in Pfam database. The databases’ details were provided in the supplementary information (Supplementary Figure S1; Supplementary Table S3). Here, we discussed the annotation outcomes obtained from three mostly referred annotation databases, i.e., KEGG, KOG and GO, covering the maximum range of DEGs discovered in our results. Further, the annotation of these unigenes was analyzed according to the eukaryotic orthologous group (KOG) classification, as presented in Supplementary Figure S2. Out of the 25 functional categories from the KOG database, the “General function prediction only” got the highest unigenes which are 14,530, followed by “Posttranslational modification, protein turnover, chaperones” and “Signal transduction mechanisms” with 3,958 and 3,828 unigenes. On the contrary, “Cell motility,” “Extracellular structures,” and “Nuclear structure” got the lowest unigenes with 20, 197 and 221 unigenes (Supplementary Figure S2).

The 49,309 unigenes were annotated based on Gene Ontology analysis and were classified into three broad categories of GO: biological process, cellular component, and molecular function (Supplementary Figure S3). The category “biological process” got the maximum number of unigenes, followed by the cellular component and molecular function categories.

The KEGG enrichment pathways annotation of unigenes was also evaluated. The 47,207 unigenes were associated with 142 pathways with ‘ko01100: metabolic pathways ', ‘ko01110: biosynthesis of secondary metabolites, and ‘ko04626: plant-pathogen interaction’, harboring the highest unigenes, i.e., 1,786, 1,754 and 1,697, respectively (Supplementary Tables S4,S5). Distinct types of alkaloids biosynthesis pathways identified were ko00950: isoquinoline alkaloid biosynthesis with 150 unigenes, ko00960: tropane, piperidine and pyridine alkaloid biosynthesis with 108 unigenes, and ko00901: indole alkaloid biosynthesis with 54 unigenes.

### 3.3 Distribution of gene expression in samples

**FIGURE 1:**
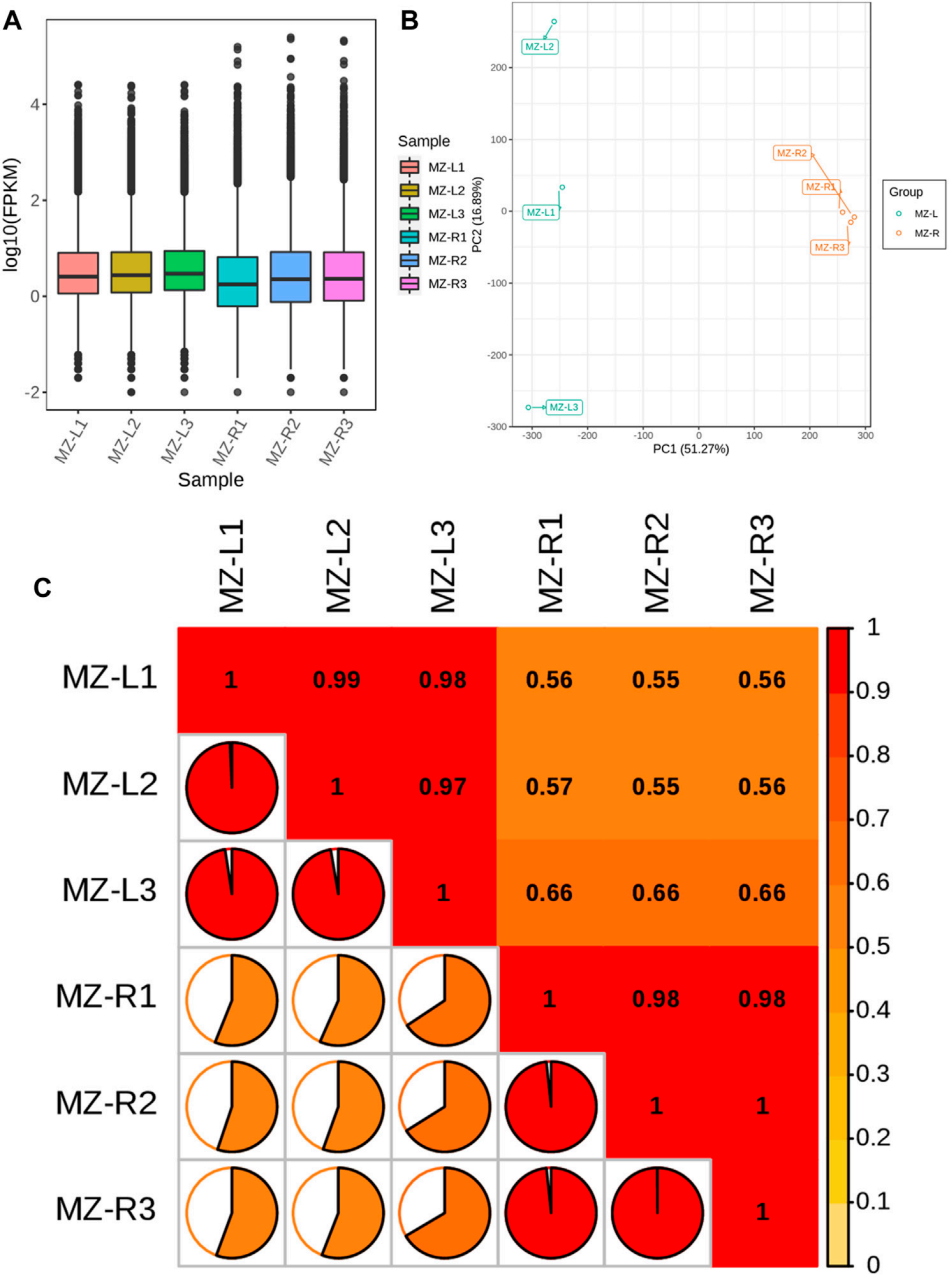
Demonstration of quality control of transcript data as **(A)** boxplots illustrating the overall fragment/kb of transcript/million mapped reads (FPKM) values for replicated samples. The abscissa depicts different samples of leaves and roots; the ordinate illustrates each sample expression’s log_2_ values for FPKM. **(B)** Principal component analysis (PCA) of the samples regarding their gene expression. The green color represents leaf samples, whereas the orange color depicts root samples. **(C)** The correlation matrix reveals Pearson’s correlation coefficient (PCC) gene expression from triplicated *V. mengtzeanum* leaves and root samples. The color gradient from yellow to red represents the range of correlation values from 0 to 1.

The detection of gene expression using transcriptome data has high sensitivity. The boxplots, principal component analysis (PCA) and Pearson’s correlation coefficient analysis revealed that the samples were alike in replicates, but sufficient variation existed among the two groups of samples. Almost 68.16% cumulative variation was covered by both PC1(51.27%) and PC2 (16.89%), with two groups of samples lying distinctly apart from each other (Figures 1A,B). Similarly, the replicates of both groups of samples showed high correlations among them but lower correlations between both groups (Figure 1C). These results provided the reliability of expression analyses conducted in the study.

### 3.4 Analysis of differentially expressed genes

**FIGURE 2:**
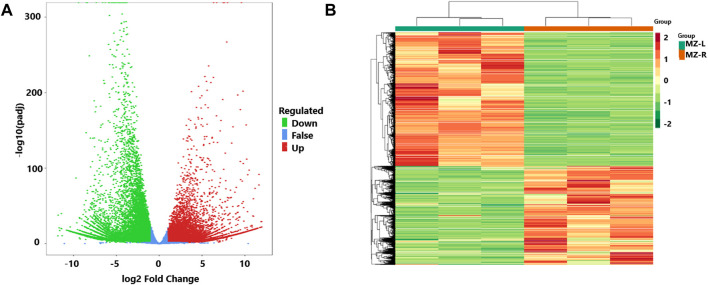
**(A)** The volcano plot of genes with differential expressions (DEGs) from leaf and root samples of *V. mengtzeanum* and revealing upregulation (red dots) and downregulation (green dots) and the non-differentially expressed genes (blue dots). The abscissa (*X*-axis) indicates the Change in gene expression multiples, and the ordinate (*Y*-axis) indicates the differential gene’s significance level. **(B)** The heatmap of FPKM values is based on differential expressions of leaf and root samples of *V. mengtzeanum*. The abscissa (*X*-axis) indicates the sample name and hierarchical clustering results, and the ordinate (*Y*-axis) indicates the differential gene and hierarchical clustering results. Red indicates high expression and green indicates low expression.

To identify the differential expression of genes (DEGs) in the leaves and root samples of the Veratrum plant, DESeq2 was utilized. The results showed 33,942 genes with differential expressions with log_2_Fold Change >1. Of them, 19,532 DEGs were found with upregulation and 14,410 with downregulation (Supplementary Table S6). The results were elaborated by plotting the volcano plots and FPKM values in the expression heatmap of identified DEGs (Figures 2A,B).

Next, the 33,942 DEGs were functionally annotated with GO and KEGG databases for leaf and root samples. The KEGG enrichment analysis revealed annotation of 15,498 DEGs for 140 KEGG pathways with the highest number of DEGs (3,194) related to ‘metabolic pathways’ followed by “biosynthesis of secondary metabolites (ko01110)” with 1,751 DEGs from ‘Metabolism class’ (Supplementary Figure S4). The DEGs involved in the biosynthesis of alkaloids and steroidal alkaloids include *AED3-like*, *3-ketoacyl-CoA synthase 10*, *MST4*, *GLB1b*, *AtDIR19*, *ethylene-responsive element binding*, *chalcone synthase 1B*, *3-O-glycotransferase 2-like*, *pathogenesis-related protein 1*, *A4U43*, *CASP-like protein 1D1*, *21 kDa protein-like*, and *sucrose synthase 1-like*.

**FIGURE 3:**
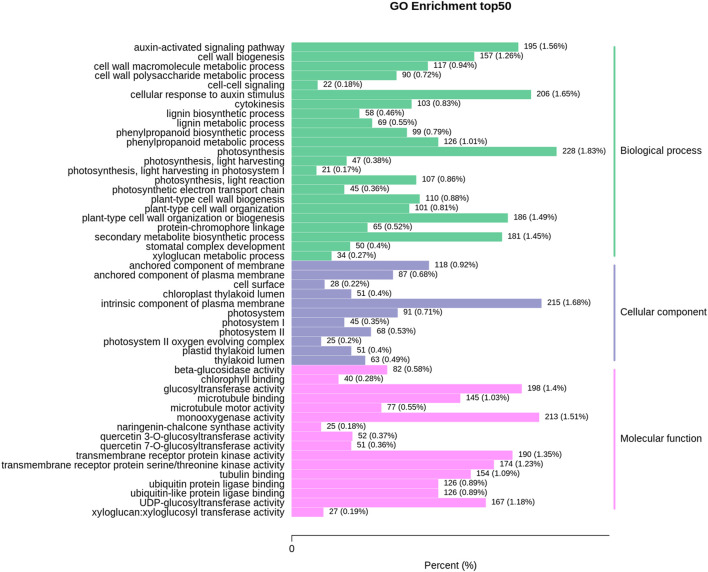
The column chart of differentially expressed genes in the leaf and root samples of *V. mengtzeanum* for topGO enrichment classification. The *X*-axis indicates the ratio of genes annotated to the total number of genes annotated to the pathway, and the *Y*-axis indicates the name of the KEGG pathway. The labels on the right side of the graph represent the classification to which the GO terms belong.

The top50 GO enrichment analysis depicted 16,421 DEGs associated with 50 biological processes, cellular components, and molecular function classes, with the highest ones (228) related to ‘Photosynthesis’ of the biological process category followed by ‘intrinsic component of plasma membrane’ from the cellular component with (215) DEGs and ‘monooxygenase activity from molecular function class with (213) DEGs (Figure 3).

### 3.5 Transcription factor annotation

As per the crucial role of transcription factors (TFs) in every biosynthetic, metabolic, cellar, and molecular function, we also discovered 897 TFs in the current study. Of 897 TFs, 377 were upregulated, and 520 TFs were downregulated in the leaf and root samples with differential expression of *Veratrum mengtzeanum* (Supplementary Table S7). From these TFs, a significant amount was involved, including *C2H2* (54), *GRAS* (51), *AP2*/*ERF* (47), *NAC* (47), *bHLH* (41), *MYB* (37), *MYB*-*related* (36), *bZIP* (34), *C3H* (33), *WRKY* (28), *B3* (22), *FAR1* (22), *GARP*-*G2*-*like* (18), and *HB-HD-ZIP* (18). Previous studies showed that many TFs were successfully isolated due to their involvement in alkaloid biosynthesis and their derivatives. Prominent TFs include *AP2/ERF*, *bHLH*, and *WRKY* for their roles in several types of alkaloids biosynthesis with responsiveness to jasmonates (JA). For example, AP2/ERF TFs that got co-upregulated more in leaves than roots with other key genes related to the biosynthesis of alkaloids include *Cluster-10506.114155*, *Cluster-10506.126873*, *Cluster-10506.18586*, *Cluster-10506.36392*, *Cluster-10506.47162, Cluster-10506.6245*, *Cluster-10506.63004*, *Cluster-10506.80539*, *Cluster-10506.84927*, *Cluster-10506.86980*, *Cluster-10506.88987,* and *Cluster-10506.92153*. Similarly, *C2H2* TFs that were co-upregulated in the sampled leaves compared to roots samples with other key genes for the biosynthesis of alkaloids and derivatives include *Cluster-10506.102281*, *Cluster-10506.111797, Cluster-10506.16970*, *Cluster-10506.5842*, *Cluster-10506.78895*, *Cluster-10506.84590*, *Cluster-10506.87434*, and *Cluster-10506.88842* (Supplementary Table S7). Likewise, other TFs such as *bHLH*, *NAC*, and *WRKY* got co-upregulated in leaf samples for alkaloids biosynthesis compared to roots samples (Supplementary Table S7).

### 3.6 DEGs involved in the alkaloid biosynthesis pathways

**FIGURE 4:**
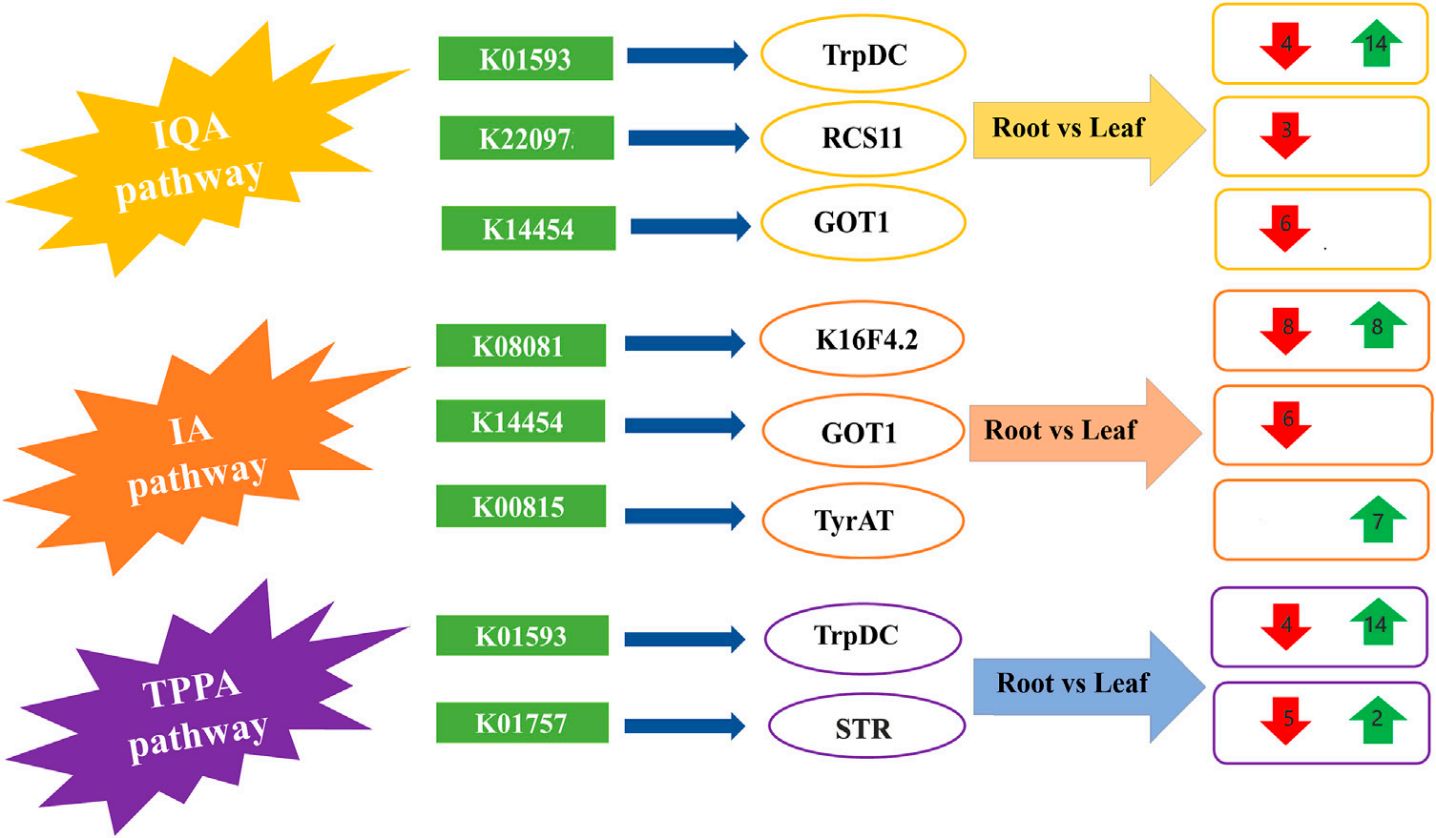
The schematic diagram for the proposed pathways of isoquinoline alkaloid biosynthesis (IQA), indole alkaloid biosynthesis (IA) and tropane, piperidine and pyridine alkaloid (TPPA) biosynthesis, along with the comparative expression of transcripts from leaf and root tissues of *Veratrum mengtzeanum*. The red and green arrows represent downregulated and upregulated transcripts in the respective pathways related to the major proteins coding DEGs.

The KEGG pathway analyses revealed that most of the DEGs involved in the biosynthesis of alkaloids include three pathways, namely and indole alkaloid biosynthesis (IA) (Supplementary Figure S5), tropane, piperidine and pyridine alkaloid biosynthesis (TPPA) (Supplementary Figure S6), and isoquinoline alkaloid biosynthesis (IQA) (Supplementary Figure S7). The number of DEGs related to IQA biosynthesis was 24 in leaves and 25 in roots, related to TPPA biosynthesis was 25 in roots and 13 in leaves, and related to IA biosynthesis was 9 in roots and 16 in leaves (Supplementary Table S8). Overall, the expression pattern of DEGs in the three pathways was equal in both leaves and roots. In previous research studies, roots were proved to be the main manufacturer of biosynthesis of alkaloids, but this study shows that both leaves and roots are significant participants in alkaloid biosynthesis. The representative pathways for IQA, IA and TPPA biosynthesis have been illustrated in schematic diagram with their enriched transcripts and related encoded proteins (Figure 4).

The IQA biosynthesis pathway included the up and downregulated transcripts for the major proteins from following KEGG annotation pathways i.e., K01593, K22097, K14454. The KEGG database showed that the L-tryptophan decarboxylase (TrpDC) protein [EC:4.1.1.28] (pathway ID: K01593) belonged to different pathways such as IQA and TPPA biosynthesis pathways with four downregulated and 14 upregulated transcripts in leaf and root tissues. The 3-O-acetylpapaveroxine carboxylesterase (RCS11) [EC:3.1.1.105] (pathway ID: K22097) and aspartate aminotransferase 1 (GOT1) [EC:2.6.1.1] (pathway ID: 14,454) were related to three and six transcripts, respectively with lower expression enriched in both IQA and IA biosynthesis pathways (Figure 4; Supplementary Table S8). A sum of 16 transcripts, eight with increased expression and eight with decreased expression related to tropinone reductase (K16F4.2) [EC:1.1.1.206] (pathway ID: K08081) were enriched in IA pathway discovered in root and leaf tissues of *Veratrum mengtzeanum*. Seven transcripts with higher expression related to tyrosine aminotransferase (TyrAT) [EC:2.6.1.5] (pathway ID: K00815) production were enriched in the IA biosynthesis pathway. In the TPPA biosynthesis pathway, seven transcripts were annotated as strictosidine synthase (STR) [EC:4.3.3.2] (pathway ID: K01757), with five transcripts showing increased and two transcripts depicting decreased expression levels (Figure 4; Supplementary Table S8).

### 3.7 Alkaloid profiling

**FIGURE 5:**
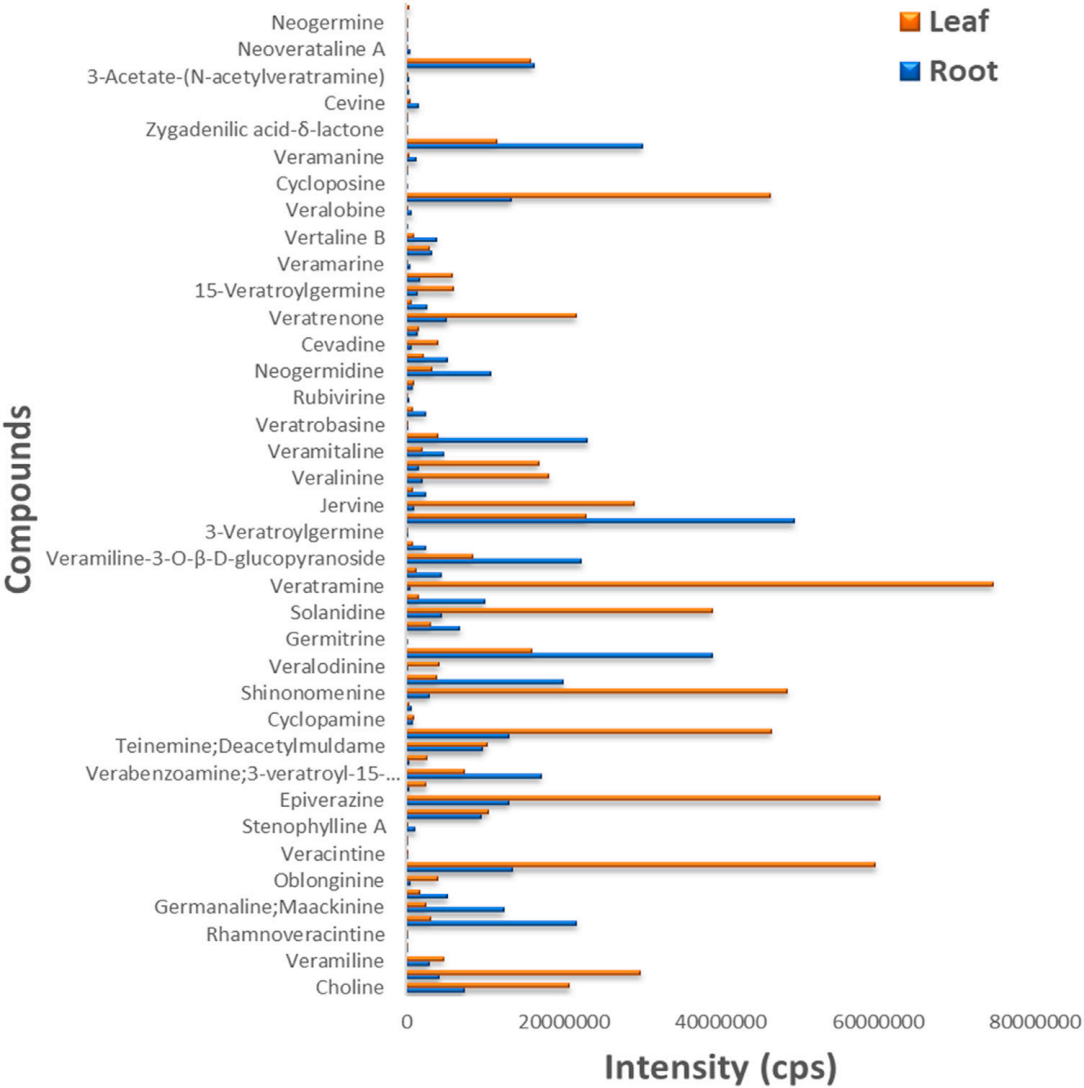
The bar plot shows 74 metabolites identified from metabolome profiling of leaf and root samples from *V*. *mengtzeanum*. Color legends on the top right illustrated leaf (brown) and root (blue) samples. *Y*-axis represented the identified metabolites and *X*-axis revealed the intensity of these metabolites’ accumulation.

The leaf and root samples were utilized to detect the differences in metabolite concentration (alkaloids) in the different plant tissues of *V*. *mengtzeanum* (Supplementary Figure S8). Six samples with three biological replicates were profiled for metabolomics using UPLC-MS/MS approach and a self-built database. A total of 74 metabolite compounds were identified, and a huge diversity exists in these identified compounds (Figure 5). The leaf samples revealed a higher accumulation of these identified metabolites in them. Out of these 74 identified metabolites, 18 highly accumulated steroidal alkaloid compounds in the leaf and root tissues of *V*. *mengtzeanum* are presented in Table 1. Of these 18 highly accumulated metabolites, 3-Vanilloylygadenine (Cmlp005885) alkaloid compound was discovered as a novel metabolite with a higher accumulation in root samples.

**FIGURE 6:**
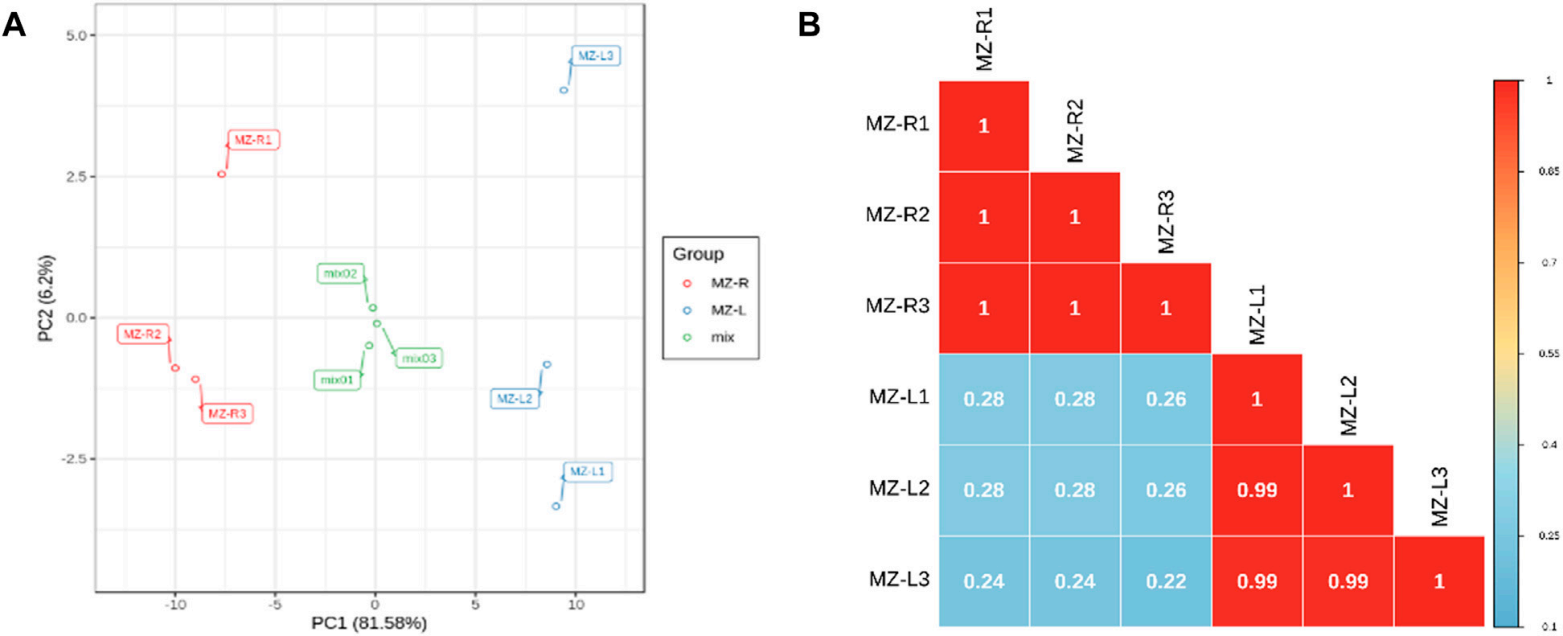
Quality control of metabolite compounds in the leaf and root tissues of *V. mengtzeanum*. **(A)** Principal component analysis revealing the leaf and root samples replicates grouping and their complete distinction from each other **(B)** Pearson’s correlation coefficients (PCC) of metabolites; each sample in triplicates and quality control mix for metabolomics. The color gradient (blue to red) bar on the right represents the correlation between the leaf and root samples of *V. mengtzeanum*.

The quality assessment of investigated samples *via* PCA and Pearson’s correlation revealed that significant differences/variations existed between the leaves and roots of *V. mengtzeanum* samples for metabolite compounds (Figures 6A,B). Further, the differentially accumulated metabolites (DAM) were studied regarding their changes between leaves and roots by plotting the OPLS-DA scores. The obtained R^2^X, R^2^Y and Q^2^ values were around 0.915, 1 and 0.998, respectively, suggesting the reliability and stability of the model used. Metabolites with criteria of variable importance in projection value ≥1 as well as top fold change ≤0.5 to ≥2 were taken as DAM between the two samples (Supplementary Figures S9A,B). A total of 55 DAM from leaves and roots of *V. mengtzeanum* samples were identified (Supplementary Table S9). It is considered that there is a higher content of alkaloid compounds in roots than in leaves, but in the current study, both leaves and roots showed a higher number of alkaloids.

### 3.8 Conjoint analyses of transcriptome and metabolome data

**FIGURE 7:**
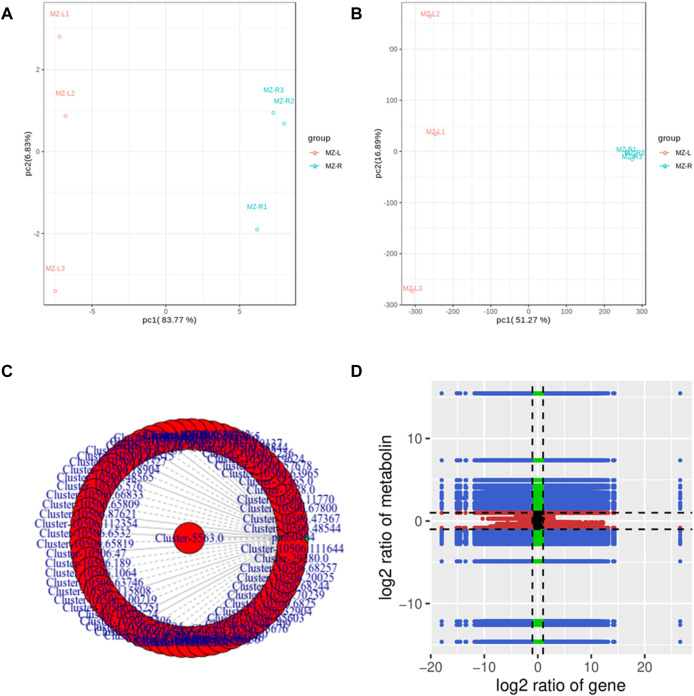
PCA and Correlation analysis of genes and metabolites detected by each differential group of samples from *V. mengtzeanum* **(A)** PCA of metabolome and **(B)** PCA of transcriptome data to visualize the differences between the sample groups of *V. mengtzeanum* in the transcripts and metabolites. **(C)** Correlation network diagram revealing a correlation between metabolites and genes. The differential genes and metabolites with a correlation value greater than 0.8 in each pathway were selected for plotting. Metabolites are marked in green; genes are marked in red. Solid lines represent positive correlation, and dotted lines represent the negative correlation. **(D)** Pearson’s correlation coefficient (PCC) of genes and metabolites through the nine-quadrant chart to show the different multiples of gene metabolites with a PCC value greater than 0.8 in each differential group, and a black dotted line, from left to right and from top to bottom, dividing the plot into 1–9 quadrants.

Both transcriptome and metabolome data of *V. mengtzeanum* were integrated and statistically analyzed to examine the relationship between genes and metabolites simultaneously coupled with other analyses such as PCA, correlation analysis, functional annotation analysis, and metabolic pathways enrichment to screen out key genes linked to metabolic pathways. Based on PCA scatterplots, the triplicated sample groups got separated and the samples from roots showed distinctly separate positions from leaf samples both in metabolites and transcriptome data results (Figures 7A,B).

Regarding differential metabolic analysis results of the current experiment, coupled with transcriptomic differential gene analysis results, DEGs and DAMs from similar groups have been simultaneously mapped by using the KEGG pathway database to understand better the relationship among these genes as well as metabolites. A total of 6,738 DEGs were discovered in association (Pearson’s correlation coefficient >0.8) with 55 metabolites, jointly controlling the regulation of alkaloids and steroid alkaloid biosynthesis in both leaves and roots. A total of 22 metabolites accumulated in the leaves and 33 in the roots (Figure 7C; Supplementary Table S10). A sum of 300 DEGs (Pearson’s correlation coefficient >0.8) was found in association with the metabolite pmb0484 (choline) in the interactive network, contributed to the production and regulation of choline content in *V. mengtzeanum* leaf and root tissues (Figure 7D; Supplementary Table S10).

### 3.9 Validation of DEGs through qRT-PCR

**FIGURE 8:**
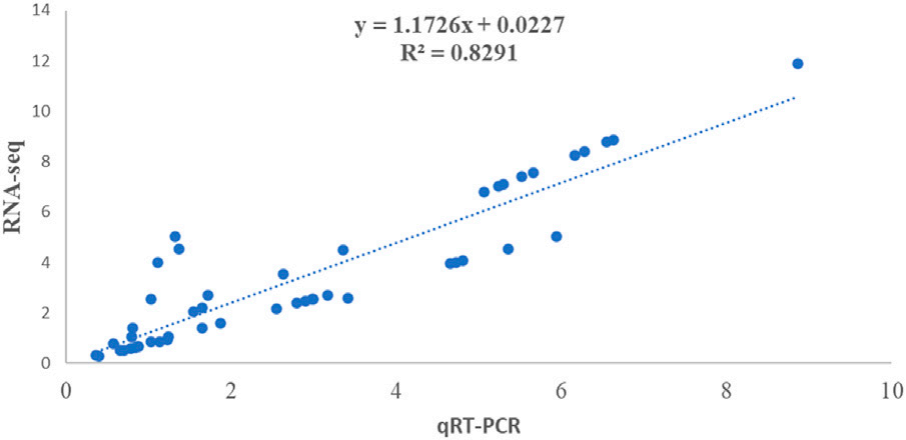
Validation of expression results using qRT-PCR representing the correlation between qRT-PCR and RNA-seq expression results for 20 selected DEGs from leaf and root tissues samples of *V*. *mengtzeanum*.

To validate transcriptome data results, 20 random DEGs involved in the alkaloid biosynthesis pathways were selected for quantitative real-time PCR analysis (Supplementary Table S11). The qRT-PCR is a world-renowned acceptable method for analyzing the expression of target genes. The results from this analysis demonstrated a consistent trend regarding the expression of selected genes in the transcription and RNA-seq data (FPKM) is consistent with the relative expression through the qRT-PCR method validating the results of transcriptome data. Furthermore, a desirable correlation coefficient, i.e., *R* ^2^ = 0.8291, was observed between RNA-seq data and transcriptome data, illustrating their positive correlation, and thus increasing the reliability of the results obtained (Figure 8).

## 4 Discussion

*Veratrum mengtzeanum* belongs to the family Liliaceae and is native to Southwest parts of China, particularly Guizhou, Yunnan and Sichuan, with high medicinal value and poisonous nature (Han et al., 2019). Almost 43 species of *Veratrum* around the globe are utilized due to their medicinal importance (Mudunuri and Nagarajaram, 2007; Han et al., 2019). The roots/rhizomes of this plant are efficient in curing different painful and inflammatory diseases and injuries (Li et al., 2019a). In the current study, we utilized both leaves and roots of *V. mengtzeanum* to detect alkaloids and steroidal alkaloids. The study indicated that leaves and roots are significant producers of alkaloids and steroidal alkaloids. It makes the plant even more valuable for the pharmacology and medicine industry. Many species of the genus *Veratrum* have been studied in the past for their medicinal significance but this species, i.e., *V. mengtzeanum* is the first ever explored regarding its medicinal importance.

Many higher plant species produced low molecular weight chemicals like alkaloids, terpenoids or phenylpropanoids. The primary function of these secondary metabolites is to provide defensive immunity to the body against pathogens (Yamada and Sato, 2013); hence, they are widely used in pharmaceutics, essential oils, flavorings, *etc.* Due to harboring biologically potential activities, alkaloids are found in 20% of plant species and are used as narcotics, poisons, pharmaceutics and stimulants (Facchini, 2001). Several researchers have recently begun isolating these secondary metabolites from intact plants after characterizing and elucidating the biosynthetic pathways of alkaloids at the enzyme level (Yamada and Sato, 2013). This species also contains many enzymes and critical regulatory pathways in producing significant alkaloids.

Alkaloid biosynthesis analysis uncovered 33,942 differentially expressed genes (DEGs) having differential expression regarding leaf and root tissue samples. Three main alkaloid biosynthetic pathways, i.e., isoquinoline alkaloid (IQA), indole alkaloid (IA), and tropane, piperidine and pyridine alkaloid (TPPA) biosynthesis pathways were identified in association with DEGs, and secondary metabolites detected from leaf and root samples. These pathways were often reported in several previous studies in plants for the regulation and production of alkaloids with pharmacological characteristics like antitumor, anti-inflammatory, analgesic, and anti-microbial ones (Rischer et al., 2006; Hao, 2019; Huang et al., 2019). The DEGs discovered include *AED3*-*like*, pathogenesis-related protein, *A4U43*, 13-ketoacyl-CoA synthase, *MST4*, *GLB1b*, *AtDIR19*, *ethylene-responsive element binding*, *chalcone synthase 1B,* 3-*O-glycotransferase 2-like*, *CASP-like protein 1D1*, *21 kDa protein-like*, and *sucrose synthase 1-like*. In previous studies, these major alkaloid-associated genes were reported to have anti-inflammatory characteristics. *AED3*-*like* gene was already discovered for alkaloid biosynthesis in microbes, i.e., *aspergillus fumigatus,* which was transferred to plants through genetic and metabolic engineering (Kutchan, 1995; Baccile et al., 2016). The DEG encoding 13-ketoacyl-CoA synthase enzyme was reported previously for antibacterial, anticholinesterase and cytotoxic-related functions in different plants (Matsui et al., 2017; Wang et al., 2017).

The gene, *A4U43,* controlling the production of Ubiquitin-like domain-containing protein, was discovered earlier from Garden asparagus (*Asparagus officinalis*) found on its Chromosome 4 and Chromosome 10 (https://www.ebi.ac.uk/). A serine-threonine kinase protein (55 kDa) encoded by identified gene *MST4* in the current study has earlier been discovered to be involved in activities related to immunity against pathological inflammations by being a member of germinal center kinase family (GCKIII) (Jiao et al., 2015) and increased growth rate with higher expression at places like thymus, placenta and blood leukocytes (Lin et al., 2001). *GLB1* gene was earlier reported for encoding proteins that participate in tolerance and resistance against abiotic stresses in rice (Ke et al., 2018; Lin et al., 2019) and for encoding allergenic seed storage proteins in common buckwheat (Sano et al., 2014). A member of the dirigent protein family *AtDIR19* was identified in the current study. These proteins were previously reported to be involved in lignification and produce a response against pathogen infections (Jin-long et al., 2012; Xu et al., 2021).

The significant proteins related to transcripts with differential expressions involved in the IQA, IA and TPPA biosynthesis pathways discovered in root and leaf tissues of *V*. *mengtzeanum* were TrpDC, RCS11, GOT1, K16F4.2, TyrAT, and STR. Some of these enzymes’ production concerning alkaloid biosynthesis are supported here by previous reports. The enzyme tryptophan decarboxylase (TrpDC) was discovered earlier in an alkaloid-producing medicinal plant *Vinca minor*, in the leaf tissues with essential functions in the indole biosynthesis pathways (Molchan et al., 2012). In *Solanum lycopersicum*, TrpDC was discovered with significant role in melatonin biosynthesis as a first step required for plant growth and development especially under stress (Pang et al., 2018). The enzyme tyrosine aminotransferase (TyrAT) was reported in earlier studies on opium poppy (Lee and Facchini, 2011) and *Prunella vulgaris* (Ru et al., 2017) for functions performed during IQA biosynthesis and a medicinal compound “rosmarinic acid” biosynthesis, respectively. The protein tropinone reductase (K16F4.2) was reported earlier from the extractions from leaf, root and stem tissues of the medicinal plant *Dendrobium nobile*; widely known for its alkaloid (pharmacological and clinical effects) production (Cheng et al., 2013) and in *Solanaceae* plants for having roles in the metabolism of tropane alkaloids (Dräger, 2006). In previous studies, strictosidine synthase (STR) was reported in plants (*Gossypium* species, *Oryza sativa* and *Arabidopsis thaliana* etc) as an essential enzyme to derive natural alkaloid compounds that have significant pharmacological effects (Cao and Wang, 2021). In rice, this enzyme showed involvement in biosynthetic pathways related to plant growth, metabolism, pollen wall formation, and another development, illustrating the leading role in cell division processes (Zou et al., 2017).

Similarly, many TFs were found for the biosynthesis of alkaloids, i.e., *AP2*/ERF, *bHLH*, *MYB*-related, *WRKY*, *C2H2*, *GRAS*, *NAC*, *bZIP*, *C3H*, *B3*, *FAR1*, *GARP*-*G2*-like, and HB-*HD*-*ZIP* from leaves and roots with mostly upregulating in the roots. The TFs, i.e., *AP2/ERF*, *WRKY*, *C2H2*, *bHLH*, and *MYB*-related, are major regulators for the biosynthesis of IQA, IA and TPPA alkaloids pathways in rice, cotton, tobacco, *Fritillaria roylei*, *Catharanthus roseus*, and *V. californicum* (Yamada et al., 2011; Yamada and Sato, 2013; Augustin et al., 2015; Van Moerkercke et al., 2015; Lu et al., 2022; Zhang et al., 2022). The first TF isolated for having a role in the biosynthesis process of alkaloids was *AP2*/*ERF* in response to Jasmonates (JA) (Yamada and Sato, 2013; Zhou and Memelink, 2016; Pan et al., 2019). These TFs were also previously isolated from tobacco that produced several types of secondary metabolites, like anatabine, anabasine, nicotine, *etc.*, in response to JA in roots (Todd et al., 2010; Sears et al., 2014). The *WRKY* TFs have already reported roles in *C*. *japonica* for the biosynthesis of alkaloids in the root tissues (Kato et al., 2007; Suttipanta et al., 2011).

The TF *bHLH* was reported in several studies homologous to *MYC2* of *Arabidopsis* that regulated nicotine alkaloid biosynthesis (Todd et al., 2010; Shoji and Hashimoto, 2011). The *bZIP* TF was reported earlier for participation in response to environmental stresses (Yamada and Sato, 2013; Yan et al., 2019). In this study, these TFs are now reconfirmed for their role in regulating alkaloid and steroid biosynthesis. Furthermore, some TFs were discovered as new in the roles of regulating alkaloid biosynthesis. For example, GIBBERELLIC ACID INSENSITIVE REPRESSOR OF *ga1-3* SCARECROW protein are regulated by *GRAS* transcriptional regulators especially in pathway related to gibberellins signaling in plants involved in several response processes particularly stress responses, environmental factors and development of plant (Pysh et al., 1999; Vera-Sirera et al., 2016) and in grapes for cold stress tolerance by regulating jasmonic acid production (Wang et al., 2021). FAR-RED IMPAIRED RESPONSE1 proteins controlled by regulators *FAR1* are involved in the light induced expression of proteins that participate in chlorophyll biosynthetic pathways (Wang et al., 2016; Liu et al., 2020). The genes encoding GARP transcription factors in plants made from G2-like as well as ARR-B proteins, are discovered with diverse functions such as circadian clock rhythm, development of roots, shoots, and chloroplast, nutrient sensing, floral transition, as well as hormonal signaling pathways and their transport (Yamada and Sato, 2013; Safi et al., 2017).

Concerning the metabolome results, many metabolites (55) were associated with the biosynthesis of alkaloids and steroid alkaloids in leaves and roots. In several previous research reports, the *Veratrum* alkaloids have been reported in other species of the genus *Veratrum* (Table 1) for their diverse pharmacological characteristics, including antitumor, anti-microbial, antihypertensive, antifungal, antirheumatic, anticonvulsants, anti-inflammatory, antiviral, antithrombosis, and antitussive effects (Dumlu et al., 2019; Li et al., 2020; Meng-Zhen et al., 2020; Melnik et al., 2022). A characteristic steroidal alkaloid jervine found in both leaves and roots of *V. mengtzeanum* in the current study has already been isolated from rhizomes of *V. album* for its anti-inflammatory and antioxidant functions against carrageenan-induced paw edema of rats (Dumlu et al., 2019; Meng-Zhen et al., 2020). This steroidal alkaloid has also been isolated from roots and rhizomes of *V. nigrum*, *V. taliense*, *V. californicum*, and *V. dahuricum* for the exhibition of its antitumor, antifungal, analgesic, and antiplatelet activity (Omnell et al., 1990; Li et al., 2012; Augustin et al., 2015; Li et al., 2019a; Zheng et al., 2019; Dirks et al., 2021). In another study, jervine and pseudojervine were extracted from *V. lobelianum* and *V. dahuricum* and reported as natural analogs of serotonin for stimulating the fibroblast growth activity for wound healing (Kupchan and Suffness, 1968; Shakirov and Yunusov, 1971; Suladze et al., 2006).

Another steroid alkaloid i.e., veratramine was isolated earlier from roots and rhizomes of *V. nigrum* (Li et al., 2012; Chandler and McDougal, 2014a), showing hypotensive effects and mild cytotoxicity for human glioma cell line SF188 (Cong et al., 2007). Veratramine, epi-verazine and verazine extracted from *V. nigrum* illustrated melanogenesis inhibition in mouse melanoma (Li et al., 2012) and from *V. maackii* var. *japonicum* evaluated for antioxidant activity (Kim et al., 2020). Veratroylgermine, germanitrine, stenophylline, germidine, Verdine, verabenzoamine, 3-veratroyl-15-methylbutyrylgermine alkaloids were also reported for their extractions from rhizomes of *V. nigrum* (Kupchan and Afonso, 1959; Li et al., 2012; Chandler and McDougal, 2014b; Li et al., 2020). They were evaluated for their antiviral activities and usage in treating epilepsy, scabies, jaundice, blood stroke, hypertension, wind-type dysentery, severe phlegm, chronic malaria, and apoplexy (Li et al., 2012; Li et al., 2020). *V. grandiflorum* plants were also reported to highly accumulate in their rhizomes the verazine and shinonomenine steroidal alkaloids (Kaneko et al., 1972; Kaneko et al., 1976; Kaneko et al., 1978; Chandler and McDougal, 2014a).

Veramidines A has been isolated from aerial parts of *V. maackii* var. *japonicum* characterized by anti-inflammatory effects (Tanaka et al., 2011; Chandler and McDougal, 2014b). Solanidine, veratramine, jervine, pseudojervine, verazine and Hosukinidine in other studies were extracted from *Veratrum* genus plants and characterized for antiplatelet aggregation, analgesic, antitumor, antifungal, anti-thrombosis, anti-inflammatory type pharmacological activities along with anti-accelerator cardiac action (Krayer, 1950; Langer and Trendelenburg, 1964; Kaneko et al., 1976; Kaneko et al., 1979; Zhou et al., 2003; Tang et al., 2010; Khanfar and El Sayed, 2013; Chandler and McDougal, 2014b; Meng-Zhen et al., 2020). In another study, ‘escholerine’ was reported as a hypotensive ester alkaloid isolated from *Veratrum* plants (Morris Kupchan and Ian Ayres, 1959). Some studies also showed the significance of veratramine as it suppresses the cell growth of HepG2 liver cancer in humans by induction of cell death through autophagy (Bai et al., 2018; Yin et al., 2020). Veramiline-3-O-β-D-glucopyranoside, stenophylline and 6,7-epoxyverdine were also isolated from rhizomes/hypogeal parts of *V. taliense* in previous studies for their cytotoxic and antifungal activities (Mizuno et al., 1990; Zhou et al., 2003; Sun et al., 2012). All these alkaloid compounds have been identified and characterized earlier from several other species of the *veratrum* genus, from their underground parts. Choline alkaloid compound was detected from conjoint analysis in the current study. Limited investigations were found in the reported literature. In previous studies, the choline metabolism has been suggested for pathophysiological roles in atherosclerosis development and the nervous system, with their resultant findings showing a significant relationship between choline and betaine with the risk of cardiovascular disease and cognitive impairment (Guasch-Ferré et al., 2017; Zhong et al., 2021). It is considered an essential nutrient with crucial roles played for the normal and maintained functioning of human body cells. The endogenous primary site for synthesis in the human body is the liver. Our body can take choline from various foods, such as phosphatidylcholine or lecithin. The uptake of choline by the brain, kidneys, placenta, liver and mammary glands is vital to sustain life (Zeisel and da Costa, 2009; Biswas and Giri, 2015). Our study reported the pharmacologically significant compounds mentioned above in this *V. mengtzeanum* species from both the above and underground parts.

## 5 Conclusion

Although *Veratrum* species are widely utilized for making traditional medicines, *Veratrum mengtzeanum* has rarely been explored based on molecular studies to reveal its pharmacological significance. It is the first comprehensive study providing genes and metabolites set involved in the biosynthesis of alkaloids and steroidal alkaloids in both leaves and root tissues. We discovered 33,942 DEGs and 55 DAM from transcriptome and alkaloid profiling of both leaves and root tissue samples. Furthermore, a variety of regulatory pathways, particularly ko00950 and genes (*A4U43*, *AED3-like*, *3-O-glycosyltransferase 2-like*, *AtDIR19*, *MST4*, *CASP-like protein 1D1*, and *21 kDa protein-like*) and significantly potential TFs (*AP2/ERF*, *bHLH*, *MYB-related*, *WRKY*, *C2H2*, and *bZIP*) were discovered for their roles in the biosynthesis of alkaloids and steroid alkaloids in leaves and roots of *V. mengtzeanum*. The alkaloid profiling revealed 18 highly accumulated alkaloid compounds with a novel alkaloid, i.e., 3-Vanilloylygadenine (Cmlp005885) in *V. mengtzeanum*. The conjoint analysis of metabolome and transcriptome data also identified a substantial number of DEGs associated with regulating alkaloid biosynthesis in leaves and roots and discovered choline as a highly accumulated secondary metabolite (alkaloid) in both leaves and roots of this species. Hence, leaves and roots can be used for the pharmacological extractions of alkaloids and steroid alkaloids.

## Data Availability

The original contributions presented in the study are publicly available. This data can be found here:https://www.ncbi.nlm.nih.gov/bioproject/816661

## Supplementary material

Supplementary Figure S1 A bar graph depicting the summary of unigenes' annotations of *Veratrum mengtzeanum* based on seven databases i.e., Kyoto encyclopedia of genes and genomes (KEGG), NCBI non-redundant protein sequences (Nr), curated protein sequence database (SwissProt), Translated European Molecular Biology Laboratory (Trembl), euKaryotic Ortholog Groups (KOG), Gene Ontology (GO), and Protein family (Pfam).

Supplementary Figure S2 Functional classification of *Veratrum mengtzeanum* unigenes according to the eukaryotic orthologous group KOG database for eukaryotes. The contents of each classification depicted the abscissa, and the number of genes is represented on the ordinate. Distinct colors of bars are to represent different classes.

Supplementary Figure S3 The GO terms classification as biological processes (orange), cellular components (green), and molecular functions (blue) based on *Veratrum mengtzeanum* unigenes from transcriptome data obtained from leaves and root samples.

Supplementary Figure S4 The column chart of differentially expressed genes (DEGs) in the leaves and root samples of *V. mengtzeanum* for KEGG classification. The X-axis indicates the ratio of genes annotated to the total number of genes annotated to the pathway, and the Y-axis indicates the name of the KEGG pathway. The labels on the right side of the graph represent the classification to which the KEGG pathway belongs.

Supplementary Figure S5 A putative pathway (ko00901) for Indole alkaloids (IA) to reveal the differentially expressed genes (DEGs) associated with the regulation of the pathway. The enzymes involved in the study involved in this pathway were boxed with blue labels showing association with both upregulated and downregulated genes.

Supplementary Figure S6 A putative pathway (ko00960) for tropane, piperidine and pyridine alkaloid biosynthesis (TPPA). The enzymes in the study involved in this pathway were boxed green tagged with downregulated genes, and the blue labels are related to both the upregulated and downregulated genes.

Supplementary Figure S7 A putative pathway (Ko00950) for isoquinoline alkaloid biosynthesis (IQA) is illustrated for associated DEGs. The enzymes in the study involved in this pathway were boxed with red labels showing association with upregulated genes, the green tagged with downregulated genes, and the blue labels are related to both the upregulated and downregulated genes.

Supplementary Figure S8 The cluster heatmap of differentially accumulated metabolites (DAMs), particularly alkaloids found in the leaves and roots of *V. mengtzeanum* exhibits a fold change of top significant (*p* < 0.05). The X-axis represents the sample names, and the Y-axis indicates the differential metabolites. Distinct colors in the heat map represent the values obtained after the normalization of the relative content of the differential metabolite, reflecting the level of their relative content (red: represents higher content, green: represents lower content). The annotation bar above the heat map corresponds to the sample grouping (leaves and roots); the note bar on the left side of the heat map corresponds to the Class of substance (alkaloids here). The correlation coefficients were used as a clustering algorithm to classify features determined by Pearson correlation based on average/means. The color gradient from orange (1.5) to green (−1.5) depicts several compounds, presented as relative fold change.
