# Supplementary figures and images for: Transcriptome and metabolome profiling of the medicinal plant Veratrum mengtzeanum reveal key components of the alkaloid biosynthesis
Source: Front Genet. 2023 Jan 20;14:1023433. doi: 10.3389/fgene.2023.1023433 (PMC9895797; doi:10.3389/fgene.2023.1023433)

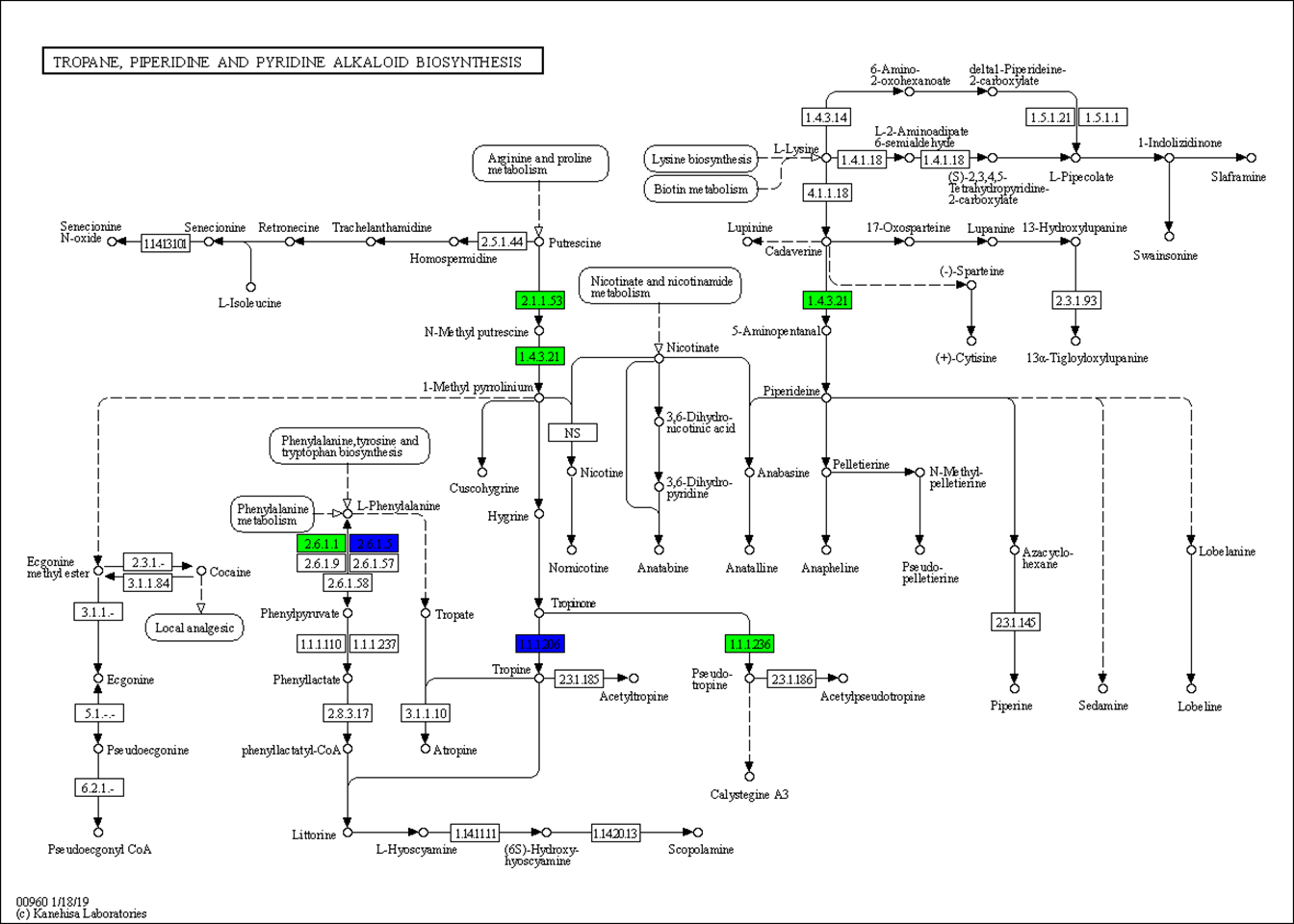

Supplement: Supplementary file 1 [file Image6.TIF]

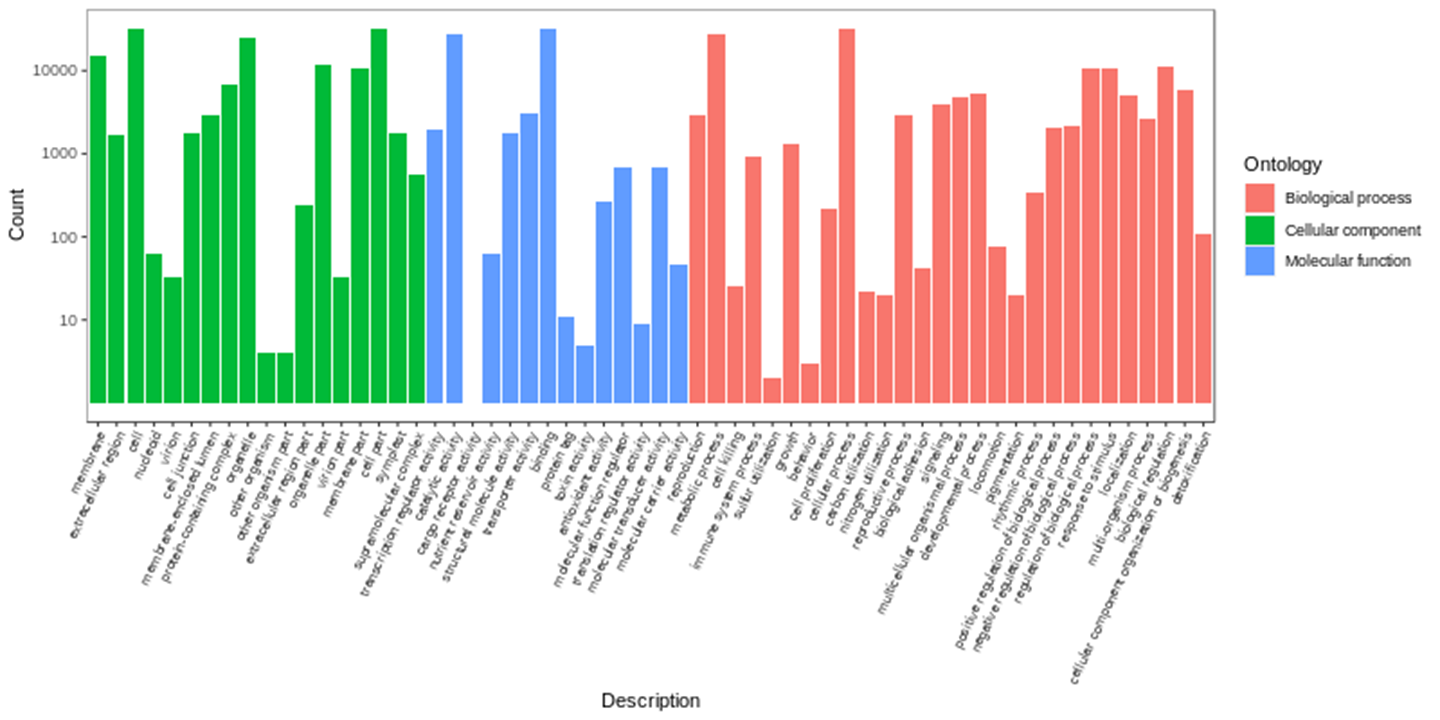

Supplement: Supplementary file 2 [file Image3.TIF]

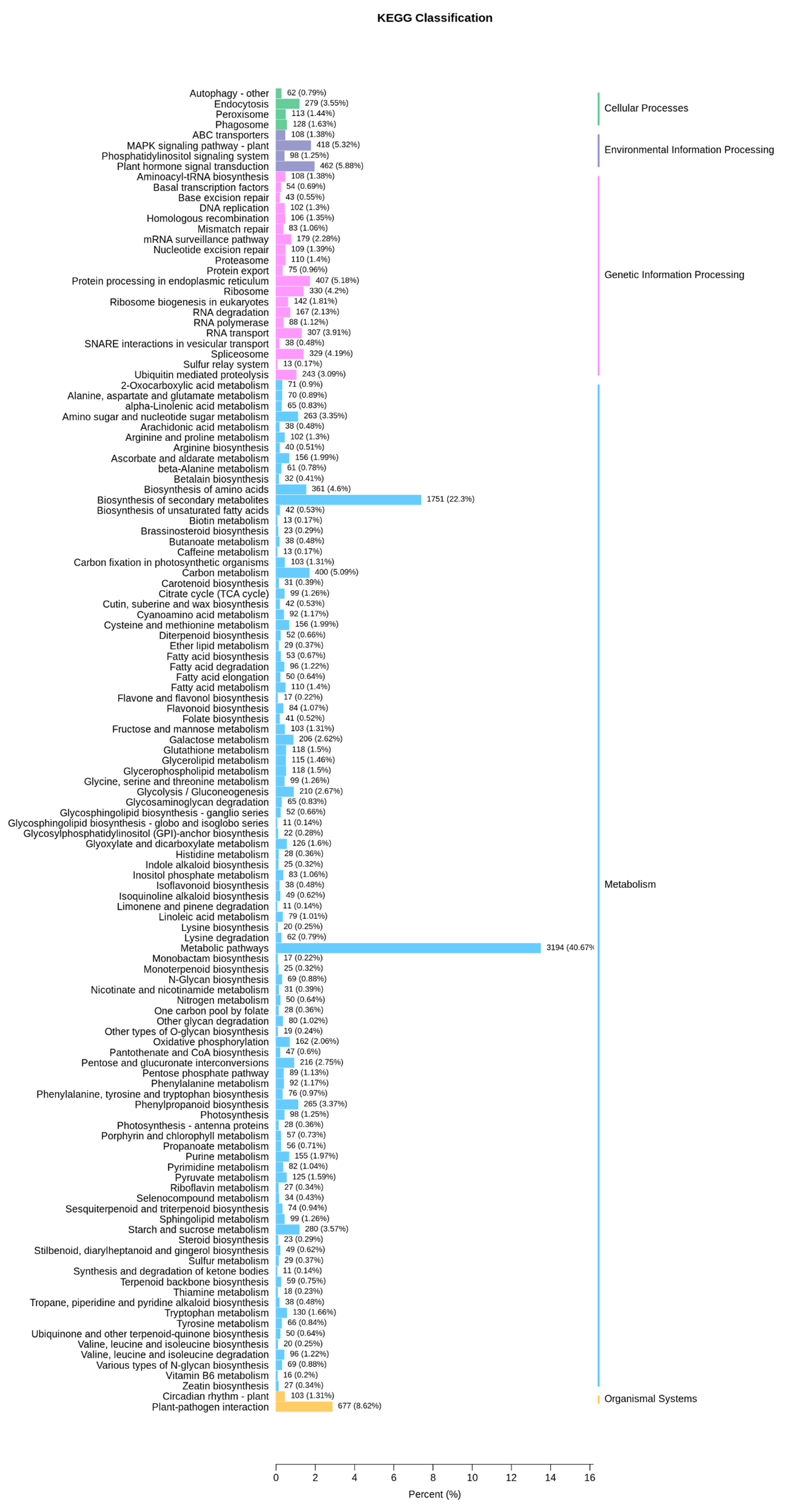

Supplement: Supplementary file 3 [file Image4.TIF]

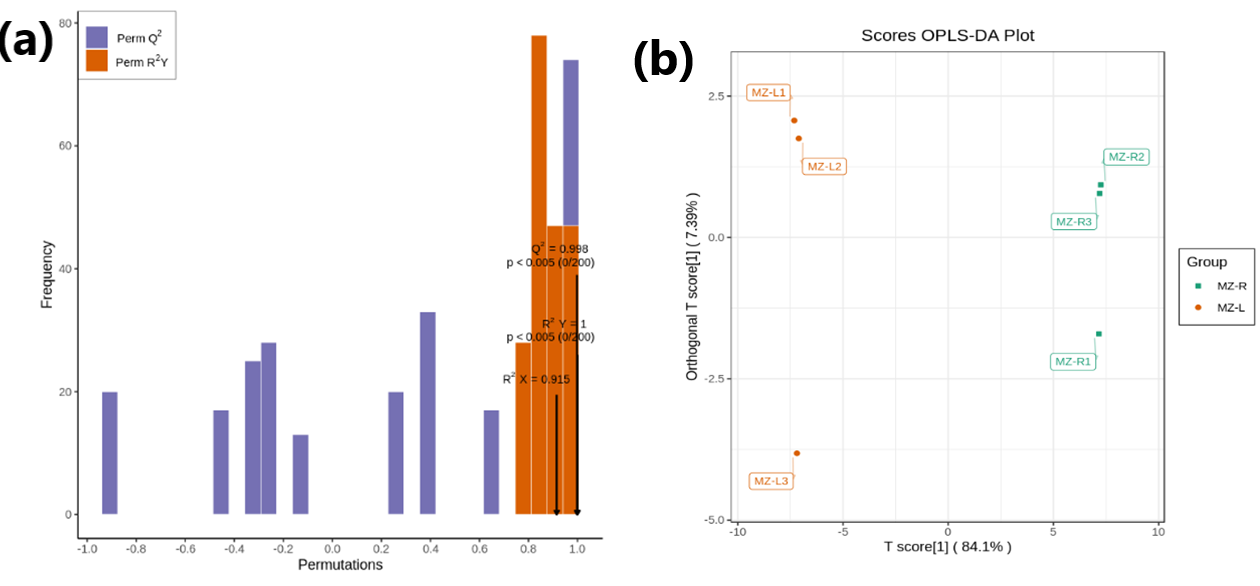

Supplement: Supplementary file 4 [file Image9.TIF]

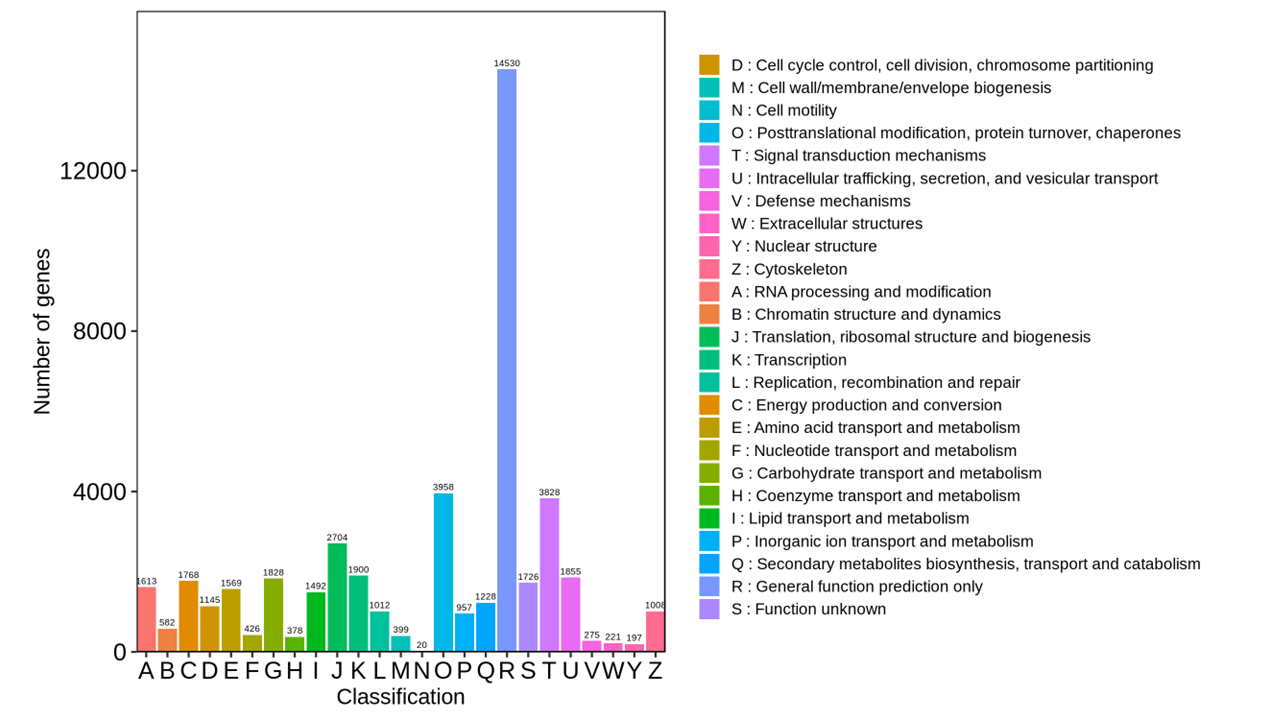

Supplement: Supplementary file 5 [file Image2.TIF]

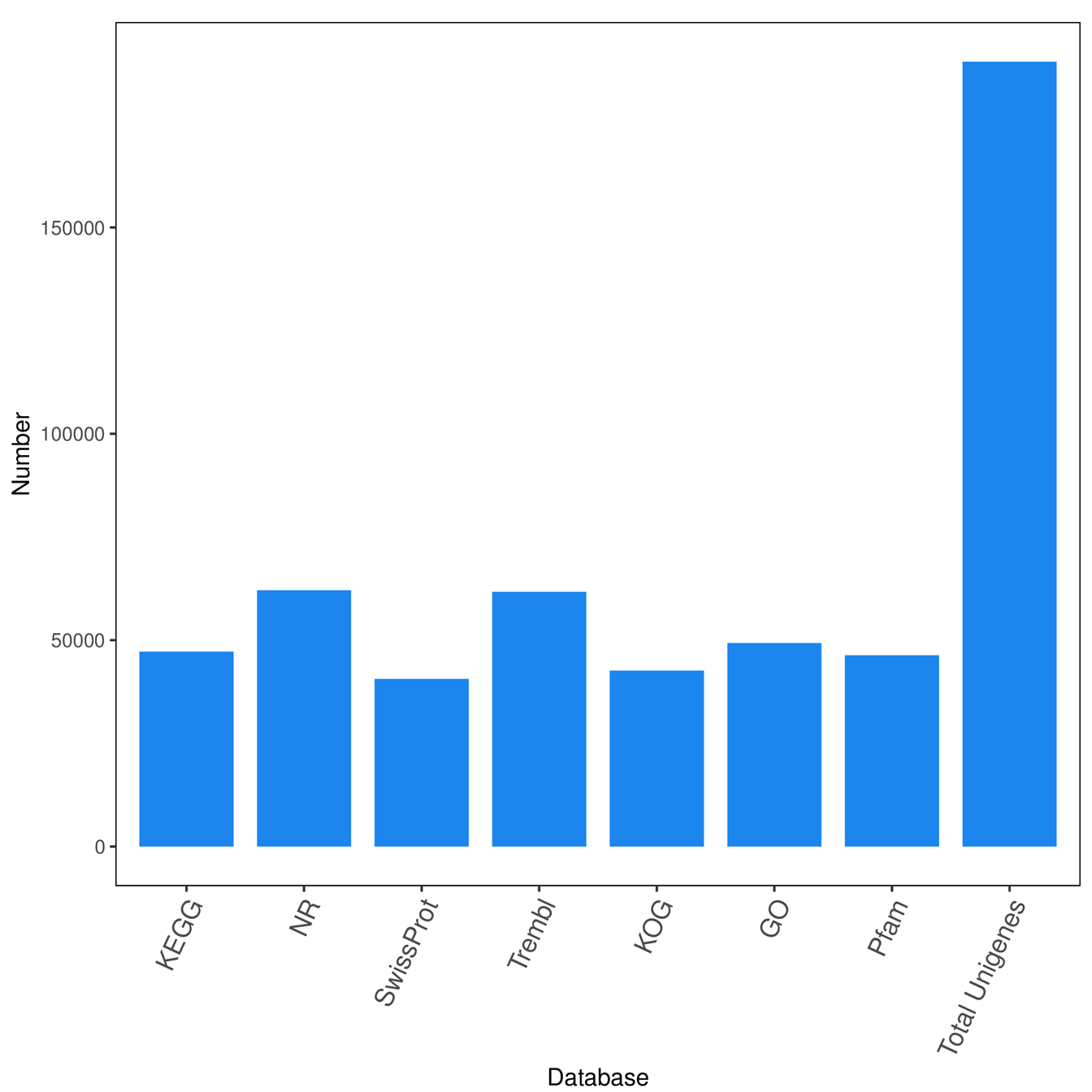

Supplement: Supplementary file 6 [file Image1.TIF]

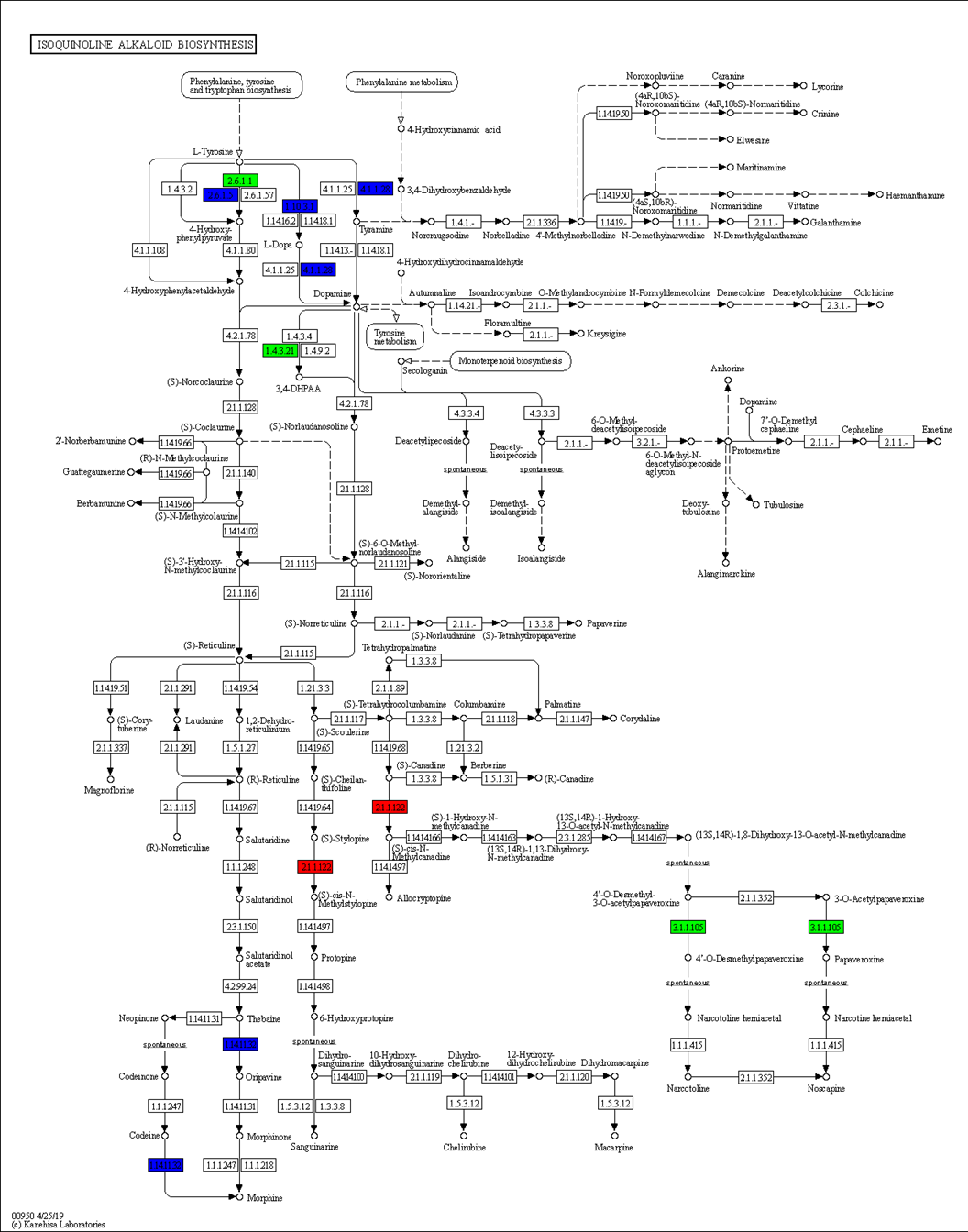

Supplement: Supplementary file 7 [file Image7.TIF]

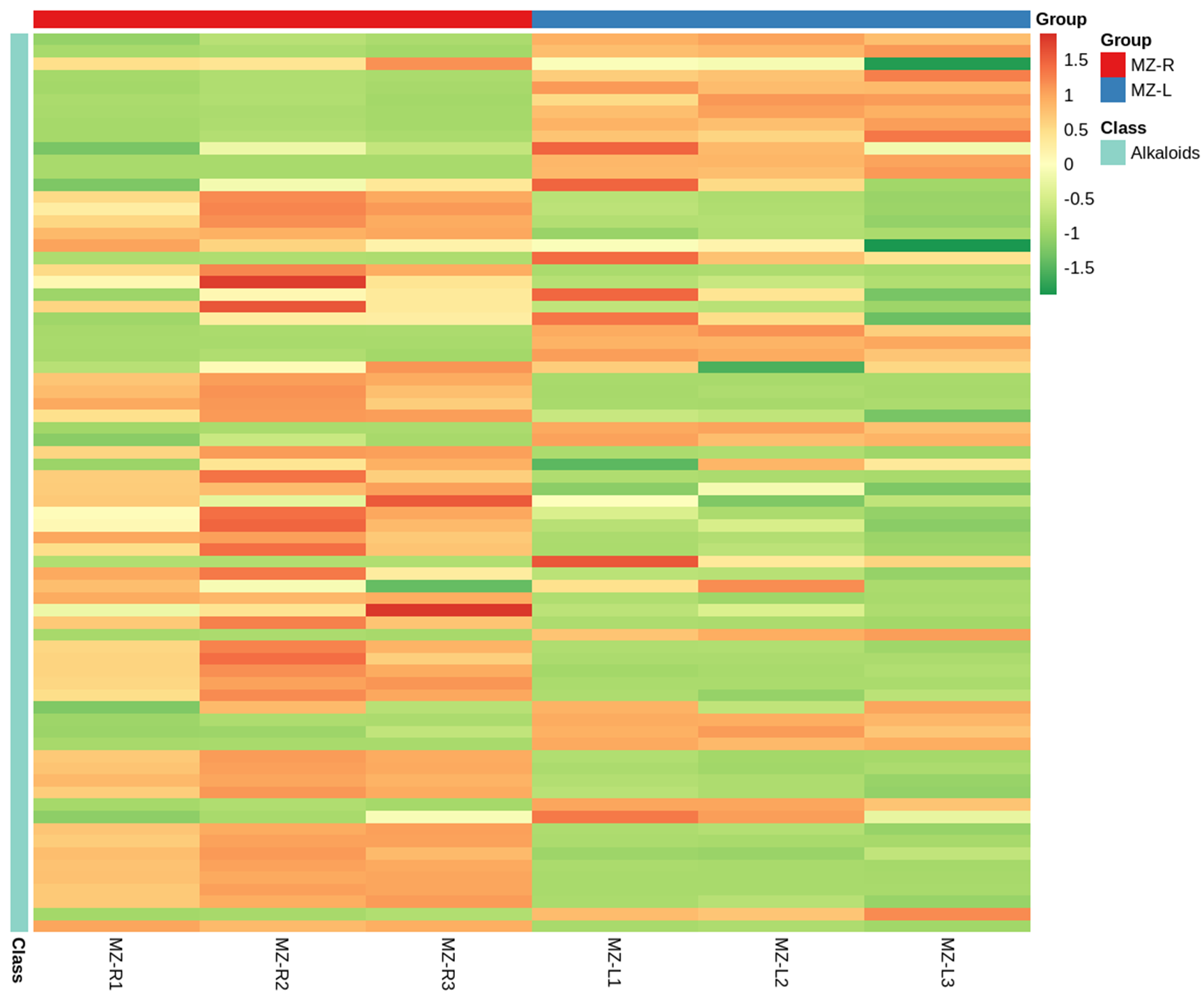

Supplement: Supplementary file 9 [file Image8.TIF]

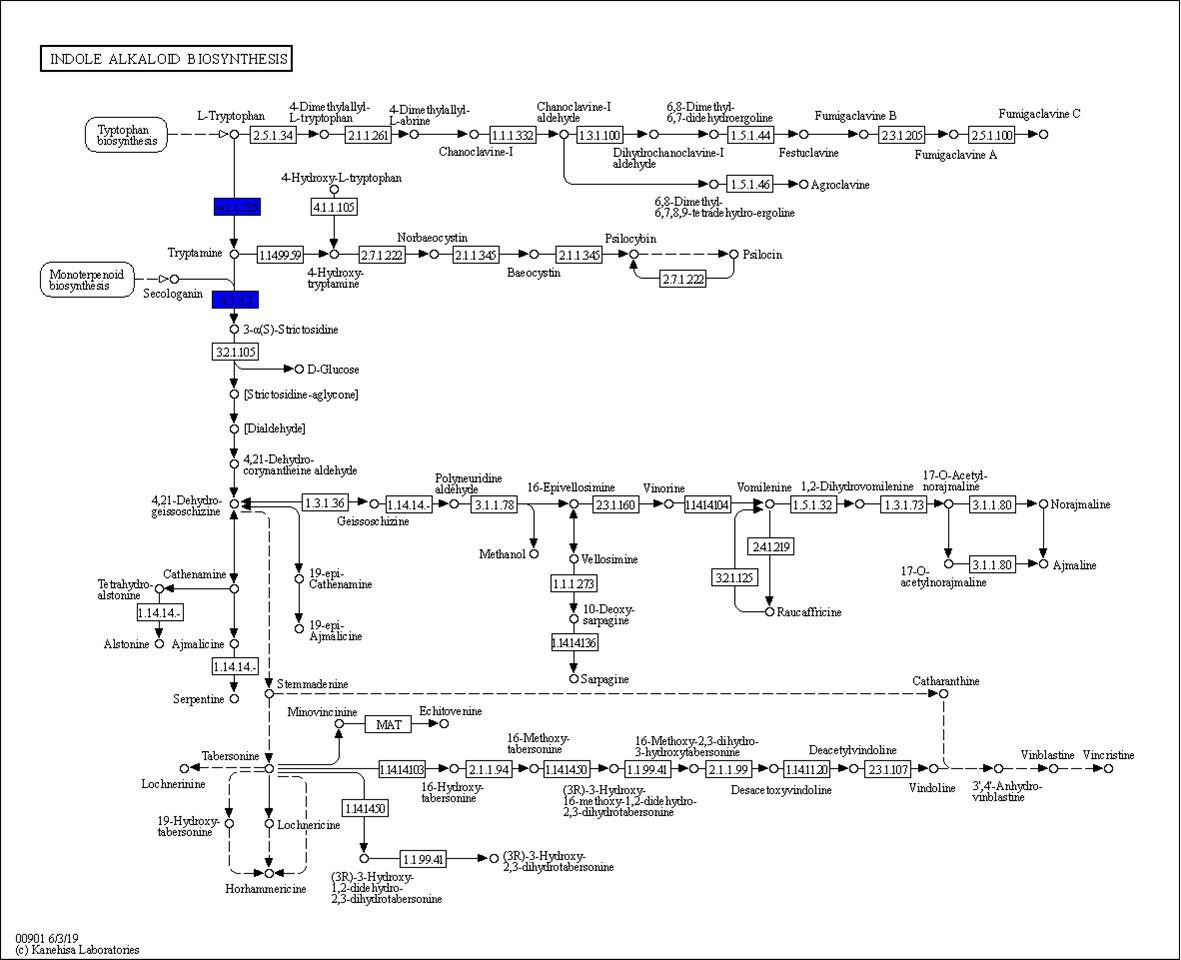

Supplement: Supplementary file 10 [file Image5.TIF]
